# Aminorex identified in horse urine following consumption of *Barbarea vulgaris;* a preliminary report

**DOI:** 10.1186/s13620-019-0153-5

**Published:** 2019-12-23

**Authors:** George Maylin, Clara Fenger, Jacob Machin, Sucheta Kudrimoti, Rodney Eisenberg, Jonathan Green, Thomas Tobin

**Affiliations:** 1 New York Drug Testing and Research Program, 777 Warren Rd, Ithaca, NY 14853 USA; 2 Equine Integrated Medicine, 4904 Ironworks Rd, Georgetown, KY 40324 USA; 3 The Maxwell H. Gluck Equine Research Center and Department of Toxicology and Cancer Biology, University of Kentucky, Lexington, KY 40546 USA; 4 Frontier BioPharm, LLC, Richmond, KY 40475 USA; 5 Department of Plant and Soil Sciences, University of Kentucky, Lexington, KY 40546 USA

**Keywords:** Horse, Urine, Brassicaceae, *Barbarea vulgaris*, Aminorex, Drug testing

## Abstract

**Background:**

Aminorex, (RS)-5- Phenyl-4,5-dihydro-1,3-oxazol-2-amine, is an amphetamine-like anorectic and in the United States a Drug Enforcement Administration [DEA] Schedule 1 controlled substance. Aminorex in horse urine is usually present as a metabolite of Levamisole, an equine anthelmintic and immune stimulant. Recently, Aminorex identifications have been reported in horse urine with no history or evidence of Levamisole administration. Analysis of the urine samples suggested a botanical source, directing attention to the Brassicaceae plant family, with their contained GlucoBarbarin and Barbarin as possible sources of Aminorex. Since horsepersons face up to a 1 year suspension and a $10,000.00 fine for an Aminorex identification, the existence of natural sources of Aminorex precursors in equine feedstuffs is of importance to both individual horsepersons and the industry worldwide.

**Results:**

Testing the hypothesis that Brassicaceae plants could give rise to Aminorex identifications in equine urine we botanically identified and harvested flowering Kentucky *Barbarea vulgaris*, (“Yellow Rocket”) in May 2018 in Kentucky and administered the plant orally to two horses. Analysis of post-administration urine samples yielded Aminorex, showing that consumption of Kentucky *Barbarea vulgaris* can give rise to Aminorex identifications in equine urine.

**Conclusions:**

Aminorex has been identified in post administration urine samples from horses fed freshly harvested flowering Kentucky *Barbarea vulgaris*, colloquially “Yellow Rocket”. These identifications are consistent with occasional low concentration identifications of Aminorex in equine samples submitted for drug testing. The source of these Aminorex identifications is believed to be the chemically related Barbarin, found as its precursor GlucoBarbarin in Kentucky *Barbarea vulgaris* and related Brassicaceae plants worldwide.

## Background

**Fig. 1:**
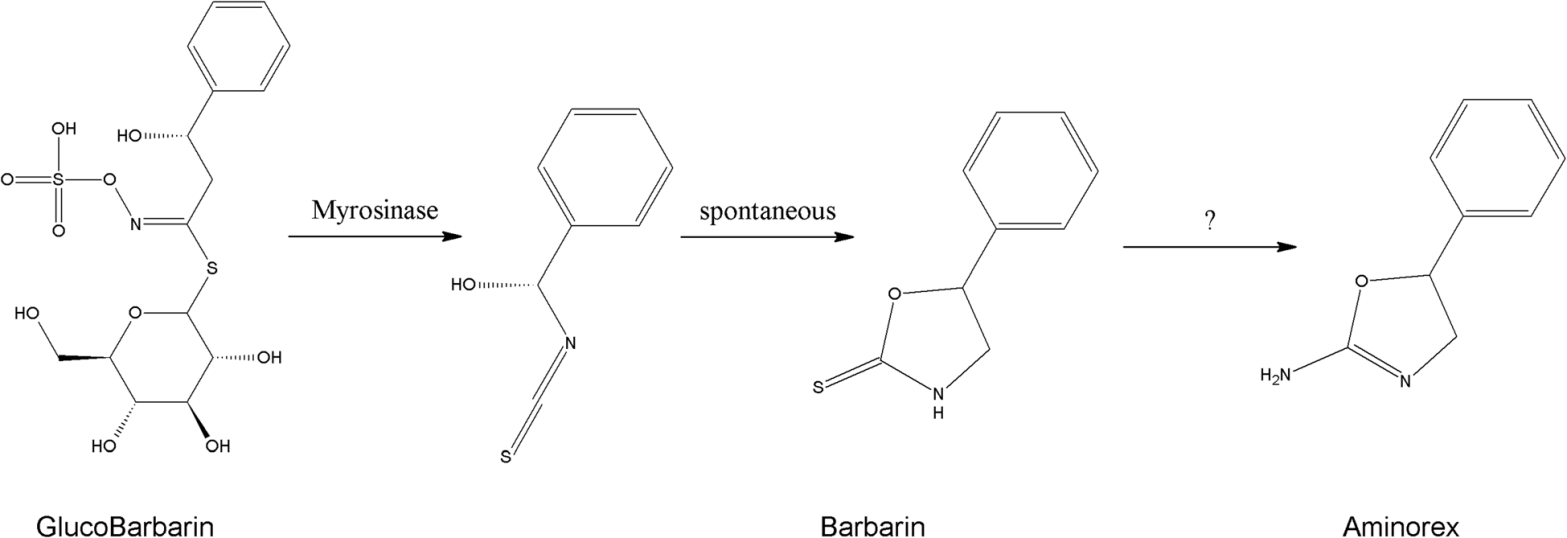
Conversion of GlucoBarbarin (via Myrosinase) to Barbarin and Aminorex structure

Aminorex, (*RS*)-5-Phenyl-4,5-dihydro-1,3-oxazol-2-amine (C_9_H_10_N_2_O, MW 162.19, Fig. 1) is an amphetamine-like anorectic and central stimulant medication and a United States Drug Enforcement Administration [DEA] Schedule 1 controlled substance. In the nineteen sixties, Aminorex was marketed as a prescription appetite suppressant in Europe but was withdrawn when it was found to be associated with a significant incidence of cases of fatal pulmonary hypertension [1].

In the United States Aminorex began to be identified and reported as “positives” in equine samples in 2004, starting in Ohio and later in Pennsylvania and Ontario, totaling 80-plus identifications [2]. Aminorex is an Association of Racing Commissioners International [ARCI] Class 1, Penalty class A foreign substance, associated with a high potential for influencing a horse’s racing performance owing to its stimulant properties. These identifications led to significant penalties against horsemen [3] but the Aminorex identifications continued, indicating that horsemen were unaware of the source of these identifications [4]. Penalty A violations include a minimum one-year suspension absent mitigating circumstances, a minimum fine of $10,000 (or 10% of the total purse) for the trainer as well as disqualification of the purse and placing of the horse on the Veterinarian’s List for 180 days for the owner. (ARCI 2017), not insignificant penalties for a professional horseman.

Levamisole, an anthelmintic and immune stimulant used in horses and livestock, was implicated as a source of Aminorex identifications in 2007 [2]. Levamisole was originally identified as an immune modulator for horses in 1997 [5], and gained popularity as an adjunctive treatment for Equine Protozoal Myeloencephalitis over the following decade. In 2009 Ho et al. identified Levamisole metabolites, Rexamino and Compound II, and concluded that these metabolites could be co-identified with Aminorex in cases where the Aminorex identification resulted from treatment with Levamisole [6]. The association of Levamisole with Aminorex led to the classification of Levamisole by ARCI as a Class 2 Penalty Class B foreign substance.

While the identification of Levamisole as a source of Aminorex led to a sharp reduction in the number of Aminorex identifications, it has not eliminated such findings, and in North America sporadic unexplained Aminorex identifications have occasionally been reported [2]. Additionally, a number of recent Aminorex identifications have been reported in English sport horses where there was no history of Levamisole administration and no co-identifications of the expected metabolic markers of Levamisole administration [7].

**Fig. 2:**
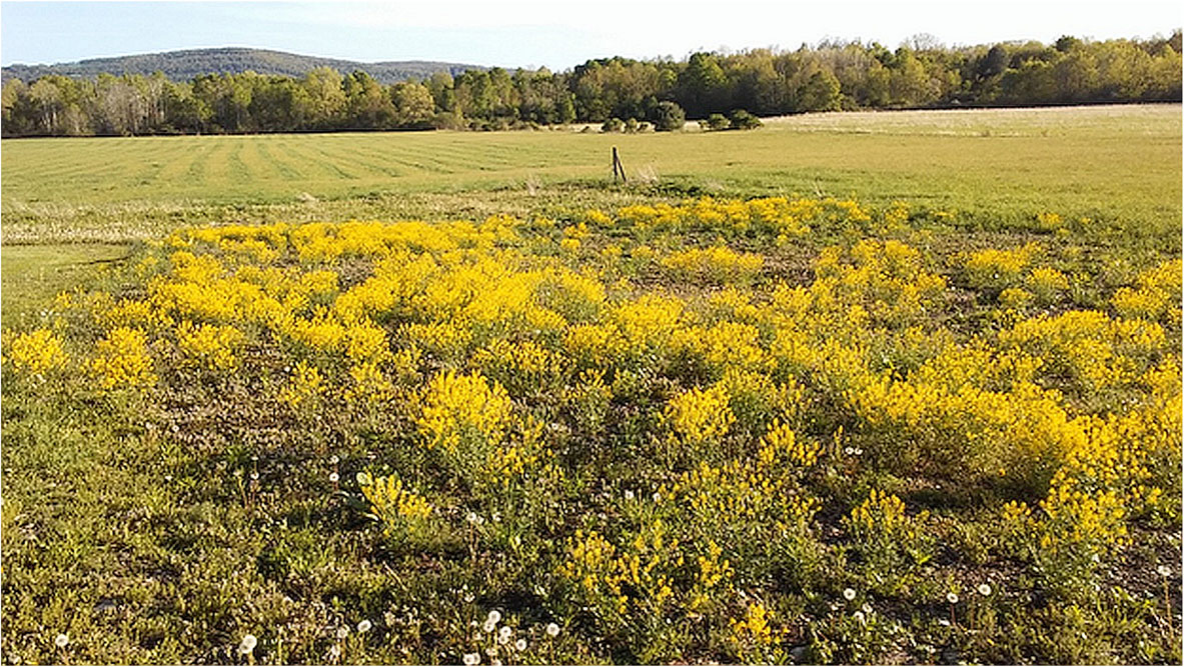
Yellow Rocket, *Barbarea vulgaris* growing in Ithaca, NY, May 21th 2018

These findings suggested an Aminorex source other than Levamisole and careful review of substances co-identified in these samples showed a number of plant-related, low molecular weight nitrogenous substances. These findings and review of the botanical literature led to the suggestion that GlucoBarbarin (Fig. 1) or its hydrolysis product Barbarin contained in certain plants and originally identified in *Barbarea vulgaris*, the plant from which the compound Barbarin was named, might be the source of these Aminorex identifications [8]. Additionally, our English colleagues reported identification of Aminorex in at least one presumably European member of the genus Barbarea [7] in the Brassicaceae family. Based on these reports, we identified a well-known member of the Brassicaceae family which flowers from late April to early May in Kentucky pasture, Yellow Rocket (*Barbarea vulgaris*, Fig. 2) and harvested this plant in flower for administration to horses, as we now report. Further, we have also synthesized and chemically purified Barbarin for use as a Certified Reference Analytical Standard and in enough quantities for equine administrations if required [9]. This reference standard was made available for use in the Aminorex related plasma and urine analyses reported in this communication.

## Results

**Fig. 3:**
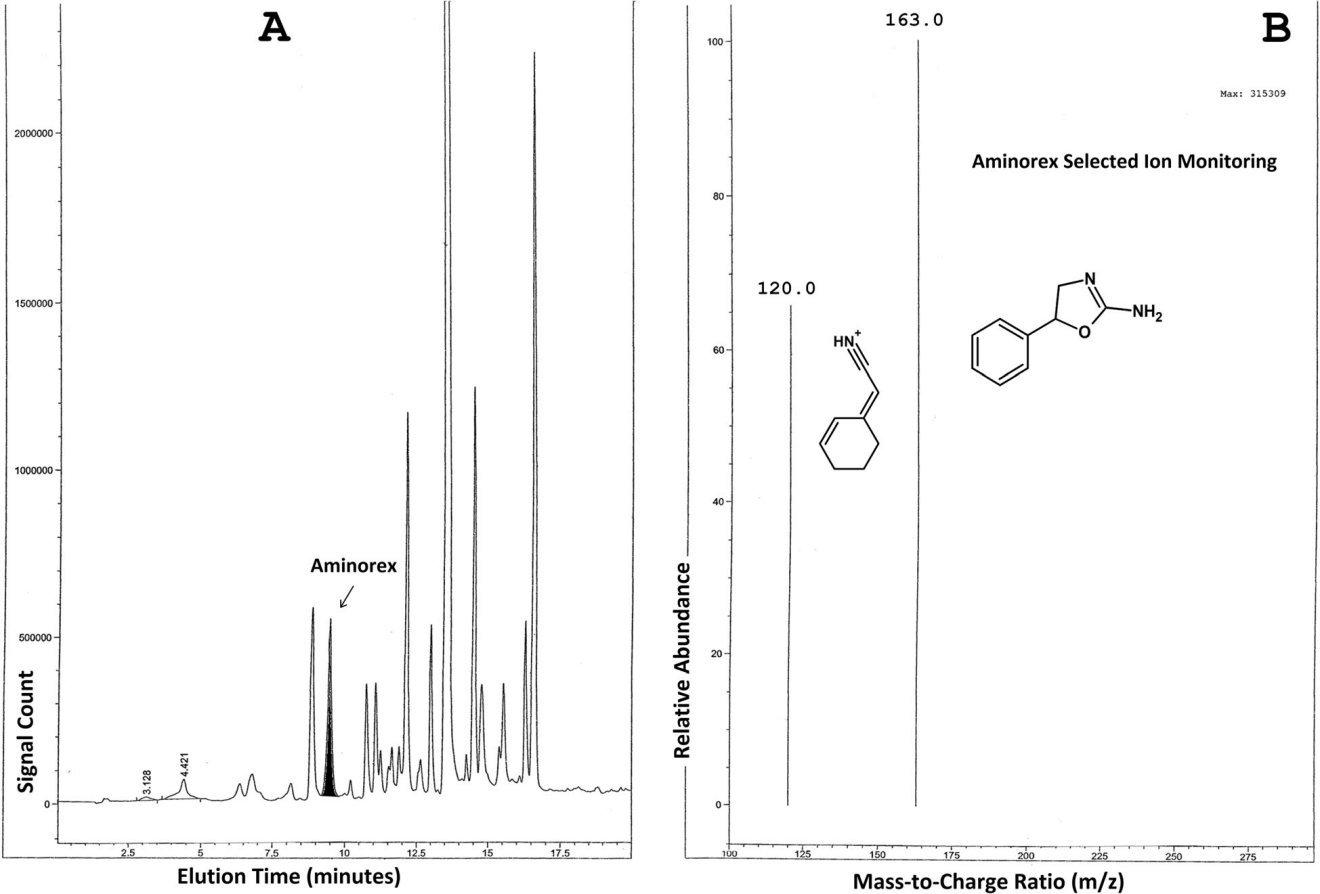
**a**: Chromatogram of a post-8-h Yellow Rocket administration urine extract scanned from 100 to 500 am with the Aminorex peak identified. Aminorex peak determined as such by the presence of the protonated ion of Aminorex at 163 am and the major daughter ion at 120 amu, as shown in Fig. 3b. **b**: The extracted protonated ions, mass 163 (Aminorex parent ion) and mass 120 (major Aminorex daughter ion) which were used to identify Aminorex in the complex urine matrix of Fig. 3a

**Fig. 4:**
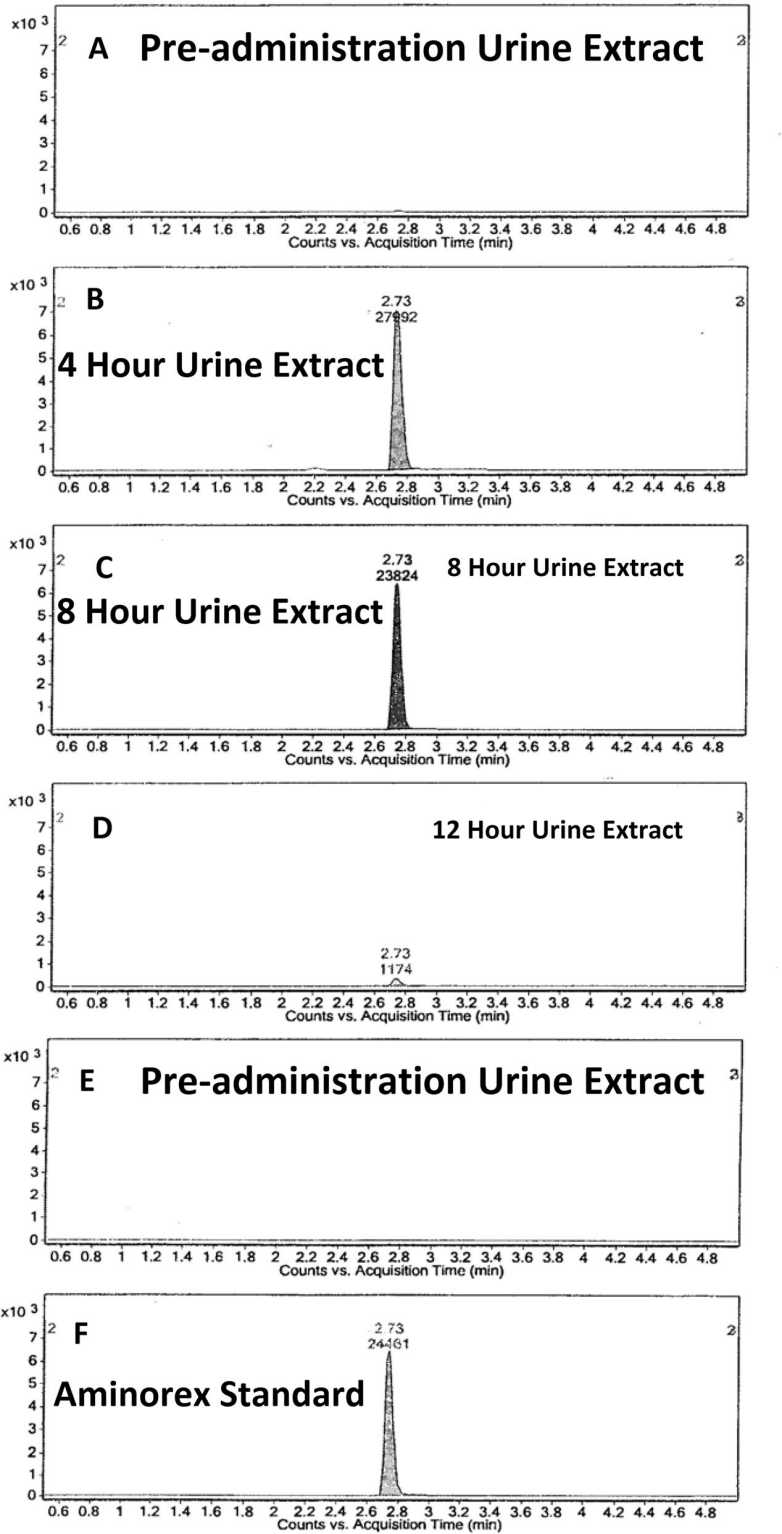
The 6470 Triple Quadrupole LC/MS data for the determination of Aminorex in pre- and post- Yellow Rocket administration urines. The transition for Selected Ion Monitoring of Aminorex was 163.1 > 103.1. **a** shows the chromatogram of the pre-administration urine extract, **b** shows the chromatogram of a 4 h post–administration urine extract, **c** shows the chromatogram of a 8 h post–administration urine extract, **d** shows the chromatogram of a 12 h post–administration urine extract, **e** shows the chromatogram of a pre-administration urine extract demonstrating no carry-over, and **f** shows the chromatogram of the Aminorex reference standard. No evidence for the presence of Barbarin in these post *B. vulgaris* administration blood or urine samples has been identified to date

Flowering Yellow Rocket plants were harvested, cleaned, and administered to horses with pre- and post-administration blood and urine test samples collected as described in the Experimental section, below. Analyses of serum and urine samples for Aminorex and Barbarin were performed in the New York Drug Testing and Research Program Laboratory using their International Association of Standardization [ISO]-17,025 validated analytical methods. Mass Spectral data obtained for Aminorex in urine after Yellow Rocket administrations are presented in Fig. 3. The chromatogram of an 8-h urine extract scanned from 100 to 500 amu is shown in Fig. 3a. Figure 3b shows the extracted protonated ions, masses 163 and 120 which were used to identify Aminorex in the complex urine matrix of Fig. 3a. The Liquid Chromatography-Mass Spectroscopy [LC/MS] data for the determination of Aminorex in Yellow Rocket administrations are shown in Fig. 4. The transition for Selected Ion Monitoring of Aminorex was 163.1 > 103.1. Panel A shows the chromatogram of the pre-administration urine, Panel B shows a 4-h urine extract, Panel C shows the chromatogram of a 12-h urine extract, Panel D shows the chromatogram of an 8 h urine extract, Panel E shows a pre-administration urine extract and Panel F shows the chromatogram of the Aminorex standard. All blood samples tested negative for Aminorex, and both blood and urine samples tested negative for Barbarin. Pre-administration urine samples were all shown to be negative for both Aminorex and Barbarin.

## Discussion

The identification of Aminorex in urine samples from horses administered *Barbarea vulgaris* is both consistent with and supports the findings and interpretations of our English colleagues [7]. Multiple members of the Brassicaceae family, including *Barbarea vulgaris,* produce GlucoBarbarin, which may be responsible for ongoing sporadic Aminorex identifications reported in post-event equine urine samples in the absence of evidence of Levamisole administration. At this time, the precise mechanism for the conversion of Barbarin to Aminorex is unclear, and it is possible that some other compound produced by *Barbarea vulgaris* is responsible. However, based on the substantial structural similarities between Aminorex and Barbarin, Barbarin is at this time the most likely candidate for the primary source of Aminorex in the Brassicaceae family.

The role of GlucoBarbarin in Brassicaceae is to serve as a Barbarin precursor. When the plant is damaged, the co-located enzyme, myrosinase, hydrolytically removes the glucose molecule from GlucoBarbarin yielding an intermediate which spontaneously cyclizes to Barbarin. GlucoBarbarin then may act like other glucosinolates in protecting the plant from potential predators [10]. Consistent with this protective function of Barbarin, the Yellow Rocket plant material was refused by the experimental horses until mixed with grass and sweet feed.

These findings are of practical significance for horse racing regulation [11], in that there are now two innocent, inadvertent, and completely unrelated sources of Aminorex identifications in post-race urine samples. The first innocent source is Levamisole, as a result of inadvertent transfer of Levamisole from its use as an anthelmintic or as an immune modulator, commonly recommended as a component of treatment for Equine Protozoal Myeloencephalitis. The second innocent source would be, as reported here, exposure to Aminorex and/or Aminorex precursors associated with inadvertent inclusion of plant fragments of the Brassicaceae family in equine feedstuffs and their subsequent ingestion by horses about to race.

Aminorex identifications in post-race urines following Levamisole administration may be confidently identified as Levamisole related if other post-Levamisole administration metabolites, Rexamino and Compound II, described by Ho et al. [12] are identified in the sample. The inability to identify these other Levamisole metabolites in the English sport horse samples initiated an investigation for other likely sources of Aminorex, leading to identification of the Brassicaceae as a likely source of post-race trace Aminorex identifications [7].

**Fig. 5:**
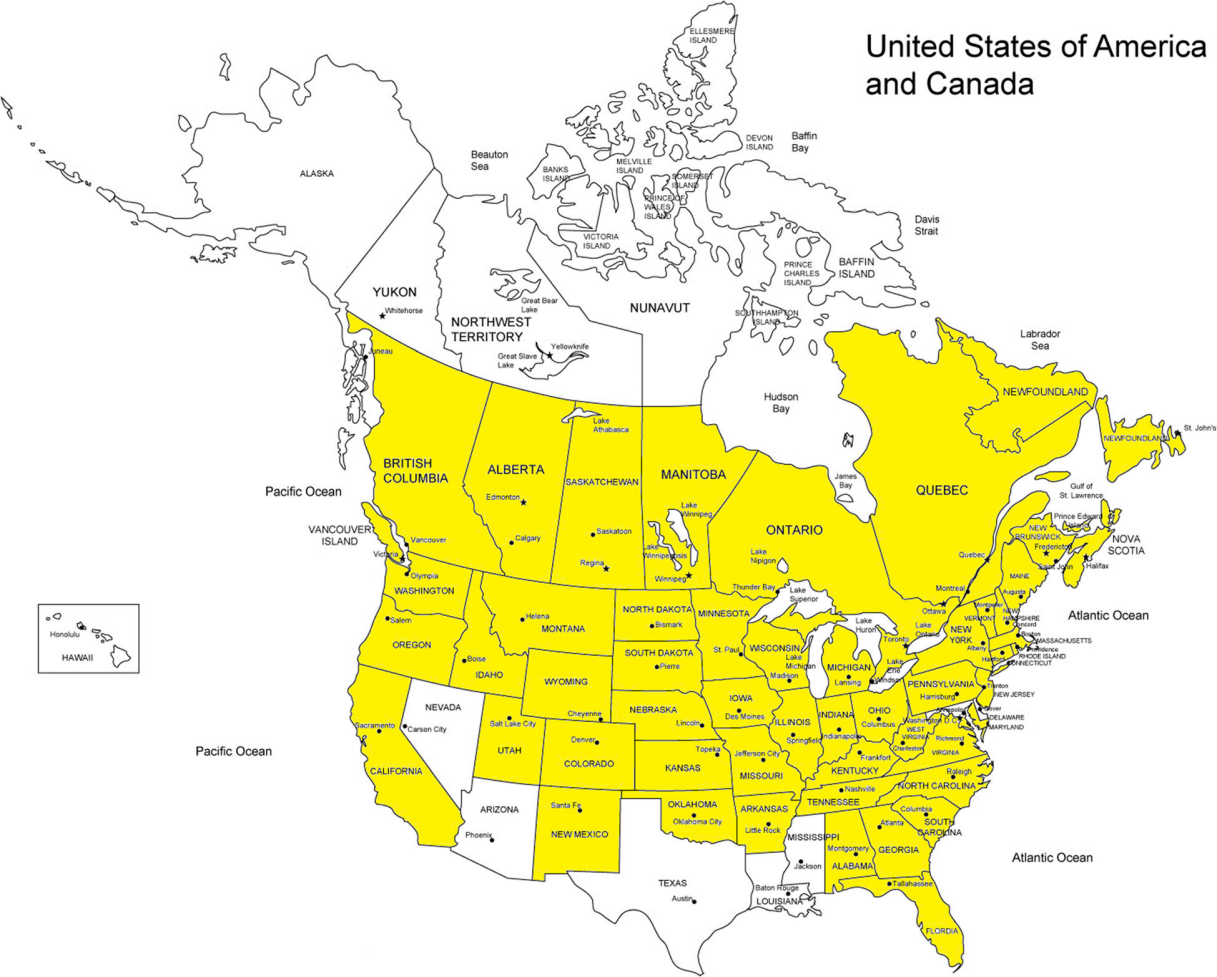
Distribution of Yellow Rocket, *Barbarea vulgaris*, in the United States; Data re-constructed from USDA reports [13]. The British Isles show similar distribution, with only parts of Scotland having none reported as of May, 2019 ([14]

Based on the wide distribution of *Barbarea vulgaris* in North America [Fig. 5] and the high level of sensitivity of post-race testing, it seems likely that a background level of sporadic inadvertent Aminorex identifications will be ongoing in American racing. One approach to addressing this issue is to identify biomarkers of exposure to Brassicaceae plants, which would allow confident identification of Brassicaceae as the source of these Aminorex identifications. At this time Barbarin, as has recently been synthesized and certified by our research program [9] would appear to be the first biomarker candidate for this role, but further research on administration of Brassicaceae and Barbarin itself to equines is likely required in this area.

A limitation of this research is that, given the worldwide distribution of Brassicaceae, we cannot at this time identify the geographic extent of this potential problem. A further concern is that the pharmacological significance of low concentration urinary Aminorex identifications is unclear. Based on the behavioral observations of Soma et al. [3] it seems that plasma concentrations of Aminorex must be at least 2 ng/mL for pharmacological effect. In fact, the brief five-minute duration of the pharmacological responses to IV administration of 15 mg of Aminorex reported by Soma et al. [3] suggests that these pharmacological responses are transient local high CNS concentration bolus responses, followed immediately by complete loss of pharmacological effect. Consistent with this interpretation, oral 15 mg administration of Aminorex produced no observable pharmacological responses. Given the fact that this orally ineffective dose produced urinary concentrations of Aminorex averaging about 75 ng/mL, it seems reasonable to suggest that urinary concentrations of Aminorex less than 75 ng/mL are unlikely to be associated with pharmacological effects.

With respect to identifying a Screening Limit of Detection (SLOD) for Aminorex in post-race urines, a cut-off / SLOD should be *“high enough to cut-off the environmental noise and low enough to stop performance enhancement”* as described in [15]. Given that a sequence of five urinary Aminorex identifications of unknown origins in Massachusetts during 2017 were all less than 20 ng/mL it would seem reasonable to set a urinary interim Screening Limit of Detection of 30 ng/mL (Fenger, personal communication, July 2019), in the absence of a significant number of data points on which to base a more complete analysis.

In presenting this interim 30 ng/mL SLOD we particularly note the unusually long terminal plasma half-life of Aminorex, 46.6 h in one horse in the Soma study. This terminal plasma half-life means that individual horses exposed to an ongoing dietary source of Barbarin will accumulate urinary concentrations over a nine-to-ten-day period, increasing the probability of attaining detectable urinary concentrations of Aminorex. Given this circumstance, it is important that reported Aminorex identifications include best good faith estimates of the concentrations of Aminorex identified. These data will allow evaluation of the contributions of geographically different environmental sources to urinary Aminorex identifications and the above interim Screen Limit of Detection can be adjusted if required, following the model presented by [16].

Other species within the Brassicaceae family may be similarly implicated as likely candidates for the introduction of GlucoBarbarin/Barbarin to animals. The plant *Reseda luteola,* more commonly known as “weld” or Dyer’s Rocket is similarly known to produce GlucoBarbarin [17]. This plant is widely distributed throughout Europe and has been introduced to many parts of the United States. Historically used as a source of yellow dye, it was also grown domestically for its sweet aromatic smell. Based on the results herein reported with respect to *Barbarea vulgaris* it is reasonable to assume that if *Reseda luteola* were to be ingested a measurable level of Aminorex might also be detectable in equine urine.

## Conclusion

In conclusion, oral administration of *Barbarea vulgaris* / Yellow Rocket plant material to two horses has resulted in urinary Aminorex identifications. The proximate chemical source of these Aminorex identifications is likely to be GlucoBarbarin/Barbarin, long-recognized components of the Yellow Rocket plant. Given these circumstances and the widespread distribution of such plants in North America and elsewhere, Yellow Rocket or related Brassicaceae plants are likely sources of sporadic low-concentration Aminorex identifications in the sports horse worldwide. Future research will focus on identifying biomarkers of Yellow Rocket or other Brassicaceae to definitively identify botanical origins for Aminorex identifications and the acquisition of enough field data to support or appropriately adjust our proposed 30 ng/mL environmental “cut-off” or Screening Limit of Detection (SLOD) for pharmacologically and forensically insignificant Aminorex identifications in equine drug testing.

## Methods

### Yellow rocket identification and harvest

*Barbarea vulgaris* plants in bloom were harvested from a central Kentucky pasture in mid-May, 2018, with the landowner’s consent and consistent with State of Kentucky plant harvesting regulations. The harvest plants were identified as *Barbarea vulgaris* / Yellow Rocket by Dr. J. D. Green of the Department of Plant and Soil Sciences of the University of Kentucky. Briefly, stems were ribbed and hairless with shiny dark leaves located in basal rosettes. Basal leaves were stalked with a large terminal lobe and smaller lower lobes. All leaves were alternate with wavy and toothed margins. The flowers were present in terminal clusters above the foliage. The identified plants were cleaned of extraneous material and shipped overnight to the New York State Drug Testing and Research Program for equine administration and forensic analysis and equine administration.

### Animal administration and sample collection procedures

The entire plant, including roots, leaves, stems and flowers were ground to a fine texture in an Oster Total Prep 10-C Food Processor. A total of 0.45 kg of wet weight processed plant material was presented to each of two horses. *Barbarea vulgaris* plant material was refused by the horses when presented alone but was readily consumed when mixed with an equivalent amount of sweet feed and fresh cut grass. Blood and urine samples were collected as pre-administration zero-time samples and then at 4, 8, and 12 h post administration.

Mature Standardbred mares weighing between 400 and 540 kg and aged 8 and 11 years were used for these administration experiments. These horses were part of the New York State Drug Testing and Research Program research herd and were in good health and monitored by experienced veterinary clinicians. Mares were maintained in their home box stalls and all sample collections and substance administrations were performed in their home stalls. Blood samples were collected by jugular venipuncture and urine samples by bladder catheterization and draining of the bladder at the indicated time intervals, as previously described [18]. Blood samples were allowed to clot, then centrifuged at 1500 g for 10 m and the serum decanted and stored at -20 °C until analysis. Urine samples were similarly aliquoted, sealed, and stored at -20 °C until analysis. All physical analyses occurred within 20 days of collection. All samples were collected under the supervision of New York State Drug Testing and Research Program personnel and transferred to the New York State Drug Testing and Research Program laboratory where they were logged in as dated and numbered research samples.

### Extraction

Aminorex was extracted from 4.0 ml urine samples adjusted with pH 9.5 carbonate buffer using 4.0 ml of ethyl acetate/hexane solvent (9: 1 v/v). The samples were mixed on a rotary mixer and centrifuged for 1 0 min each. The organic phases were transferred to concentration tubes and evaporated to dryness in a 50 °C water bath under nitrogen flow. The residues were reconstituted in methanol for LC/MS analysis.

### Liquid chromatography/mass spectrometry

An Agilent Technologies 6150 B Quadrupole LC/MS System and an Agilent 64 70 Triple Quadrupole LC/MS System in positive ionization modes were used for screening and confirmatory analysis respectively. Each instrument used 1290 Infinity Auto Injectors and thermostated column compartments. The 6150B used an Agilent Eclipse AAA, 3 × 100 mm liquid chromatography column. The LC gradient consisted of 100% A (100%/0.1%formic acid) for 5 min and then a linear gradient to 100% B (90% methanol/10% 0.1 formic acid) at 20 min at a flow of 0.4 mL/minute. Column compartment was 40 °C. The 6470 Triple Quadrupole used an Agilent Poroshell EC-120, 3.0 × 100 LC column. An LC gradient of acetonitrile/formic acid in the following composition was used: (90% formic acid/10% acetonitrile) for 1 m, (5% formic acid/95% acetonitrile) for 4.5 min and (90% formic acid/10% acetonitrile) at 5 min. Flow rate was 0.4 ml/minute and column temperature was 50 °C. Limit of Detection was 1 ng/mL and urinary concentrations of Aminorex were estimated to be approximately in the range of 10 ng/ml. No Aminorex was detected in the post-administration plasma samples, and no Barbarin was detected in either the plasma or urine samples.

## Data Availability

The datasets used and/or analyzed during the current study are available from the corresponding author on reasonable request.

## Abbreviations

ARCI: Association of Racing Commissioners International
DEA: Drug Enforcement Administration (USA)
IACUC: Institutional Animal Care and Use Committee
ISO: International Organization for Standardization
LC/MS: Liquid Chromatography/Mass Spectroscopy
SLOD: Screening Limit of Detection
